# Treatment-Seeking Behavior Among Male Civil Servants in Northeastern Malaysia: A Mixed-Methods Study

**DOI:** 10.3390/ijerph17082713

**Published:** 2020-04-15

**Authors:** Pathman Arumugam, Tengku Alina Tengku Ismail, Aziah Daud, Kamarul Imran Musa, Noor Aman A. Hamid, Shaiful Bahari Ismail, Zakiah Mohd Said

**Affiliations:** 1 School of Medical Sciences, Health Campus, Universiti Sains Malaysia, Kubang Kerian 16150, Kelantan, Malaysia; pathman_19@hotmail.com (P.A.); aziahkb@usm.my (A.D.); drkamarul@usm.my (K.I.M.); na.hamid@usm.my (N.A.A.H.); shaifulb@usm.my (S.B.I.); 2 Family Health Development Division, Ministry of Health Malaysia, Putrajaya 62590, Malaysia; drzakiahms@moh.gov.my

**Keywords:** treatment-seeking behavior, men’s health, civil servants, healthcare utilization

## Abstract

*Background*: Men’s health in Malaysia is slowly gaining more attention, but minimal research has examined how Malaysian men behave and seek treatment. While few studies have investigated men’s treatment-seeking behavior (TSB), those that have been conducted seem to be inconclusive and tend to yield contradictory findings. *Objectives:* This paper aims to determine the proportion of inappropriate TSB and to explore in-depth treatment-seeking behavior among male civil servants in northeastern Malaysia. *Methods*: This paper adopted a mixed-methods approach, specifically a concurrent parallel study design. A quantitative study using a self-administered questionnaire was performed to identify the proportion of appropriate and inappropriate TSB among male civil servants in northeastern Malaysia. Concurrently, a qualitative study was conducted involving six focus group discussion sessions, and the results of both parts were integrated to provide a detailed explanation of TSB among the participants. *Results*: A total of 381 participants were involved in the quantitative study, yielding a response rate of 94.8%; 246 (64.6%) engaged in inappropriate TSB. Some of the reported morbidities among the participants were hypertension (26.5%) and diabetes mellitus (26.2%). From the qualitative study, a main theme related to TSB emerged with several sub-themes, which were health literacy, stage of seeking treatment, preference for alternative treatment, perceived threat of illness, self-treatment, and the influence of family members and others. *Conclusions*: TSB among male civil servants in northeastern Malaysia is poor, and the factors contributing to it are multidimensional. This study has provided new valuable evidence on men’s TSB in northeastern Malaysia. The findings can be used to facilitate and improve current policies and the implementation of men’s health services throughout the country.

## 1. Introduction

Treatment-seeking behavior (TSB) is defined as a sequence of remedial actions which are influenced by many factors that individuals undertake to rectify perceived ill health [1]. In other studies, TSB has been defined as activity undertaken by individuals who perceive themselves as having health problem or to be ill for the purpose of finding an appropriate remedy [2]. The factors influencing a person’s TSB can include socio-demographic factors, individual factors, healthcare provider factors, psychological factors, socio-cultural and family factors, situational factors, and marketing factors [3]. Men’s TSB has always been thought to be a complex phenomenon, as men are consistently stereotyped as being reluctant to seek treatment when struck by illness [4]. To date, studies regarding men’s TSB and its associated factors remain limited. Based on the literature, there is growing evidence based on gender-specific illnesses which shows that men often have delayed TSB when they become ill [5]. TSB in Malaysia was identified based on gender-specific illness for both men and women. For women, TSB was mostly based on diseases related to cancer, such as breast and cervical cancer [6,7]. For men, TSB was identified based on three main areas, including were sex-related illness, chronic diseases, and infectious diseases [8]. Sex-related illnesses were conditions such as erectile dysfunction, premature ejaculation, and diseases related to same-sex sexual relationships. Chronic diseases were mostly related to diabetes mellitus, while infectious diseases were diseases such as gonorrhea and HIV [9].

According to Kapur [10], there is consistent evidence that women consult primary health care (PHC) more frequently than men. Women seem to consult PHC twice as much as men, and men consult PHC only late during the illness’s progression. For example, men tend to seek help at a later stage in cancer compared to women, who typically seek treatment at an early stage. Many studies have investigated men’s health, but very few have been conducted in Malaysia, especially with regards to TSB [10]. Based on the literature, most studies on men’s health in Malaysia have revolved around the areas of smoking, gender-specific illnesses such as sexual health issues, and diseases related to sexually transmitted diseases (STD). In the few available studies, data seems to be contradictory as well. 

While few studies on TSB among men were conducted in the past, numerous studies were performed among the general population and among specific groups as well, such as adolescents and young adults. In general, TSB was found to be better in women compared to men. In countries such as Bangladesh, Pakistan, and other countries with poor socioeconomic backgrounds, men seem to have a better TSB because they are the sole decision-makers at home. Women have no power at home, and it is up to the husband to allow their wife or children to seek treatment [11,12].

According to a study conducted in Iran, 68% of the population had inappropriate TSB [3]. Another study performed among men in Uganda stated that almost 63% of its population had poor TSB and denied free voluntary medical checkups [4]. According to another study executed in Karachi, 22% of the public had poor TSB and will never visit a healthcare facility [13]. Some of the common factors related to inappropriate TSB were a lack of support from family members, distance from a healthcare facility, attitudes of healthcare workers, sociodemographic characteristics such as age and occupation, cost of care, cultural beliefs, and the influence of friends. Acknowledging the limited research on TSB among men in Malaysia, this study provides evidence regarding men’s TSB and helps develop better guidelines focusing on a holistic approach to men’s health in the future. The aim of this paper was to identify the proportion of inappropriate TSB and further explore TSB experiences among male civil servants in northeastern Malaysia. This study highlights important findings to facilitate and improve current policies and the implementation of men’s health services in Malaysia. 

## 2. Methods

### Study Design and Selection of Participants

The study design implemented was a concurrent parallel mixed methods design which consisted of a quantitative and qualitative portion. Both parts were administered concurrently and were independent of each other. Findings from both parts were analyzed separately, and the findings were merged in the discussion. The rationale for implementing this study design was to gain more knowledge regarding TSB among the participants. It was also intended to add value and additional information to the study. This paper focused on the proportion of inappropriate TSB and emphasized the qualitative exploration of TSB experiences among men.

For the quantitative portion, the sample size was calculated using a single proportion formula. Based on the proportion of men having inappropriate TSB (68%) [3], with 95% confidence in the estimate and precision of 5%, the required sample size after accounting for the 15% non-response rate was 384. The study commenced in one of the states in the northeastern part of Peninsular Malaysia. All the districts in the state were involved. Six government agencies were pre-determined, which fulfilled the criteria of having branches in all the districts, as well as sufficient male staff with available list names of all the staff. Participants who were civil employees for at least one year, had suffered an acute illness in the past six months, and were available at the workplace during data collection were selected using simple random sampling. For the qualitative portion, data was gathered using a focus group discussion (FGD) method. The sample size was not pre-determined, but it was based on data saturation where no new themes emerged after six FGD sessions were conducted. Each FGD session consisted of about six to eight participants, and participants were conveniently chosen from the selected government agencies who fulfilled all inclusion and exclusion criteria. 

Ethical clearance for this study was obtained from the Research and Ethics Committee, School of Medical Sciences, Health Campus, Universiti Sains Malaysia (USM/JEPeM/17110602).

## 3. Research Tools

Two main tools were used in this study. A pre-validated TSB questionnaire was used for the quantitative part, and an interview guide was used for the qualitative part. The TSB questionnaire was adapted from a study conducted in Iran [3] and translated into the local language, namely, Malay. 

The questionnaire translation process from English to Malay was executed using the forward-backward translation method [14], which entails forming an expert committee, forward translation, backward translation, and pilot testing. All forward and backward translation was performed by four independent translators who were equally fluent in English and Malay. Two translators were public health physicians, and another two translators were language experts in English and Malay language. The expert committee consisted of four public health physicians and one statistician, who went through all translations and came to an agreement on all discrepancies, misunderstandings, and unclear wordings. Once all corrections were complete, the committee reached a consensus, and a pilot study was conducted among 30 male participants. Over 80% of the participants agreed that the questionnaire was easily understandable, relevant to the topic, and highly presentable. Content validity was also assessed to determine the content validity index (CVI). Five healthcare providers classified each of the items as irrelevant items (scored as 1), somewhat relevant items (scored as 2), mostly relevant items (scored as 3), and extremely relevant items (scored as 4). The CVI for these items was 0.8.

For this paper, the TSB domain was analyzed; this analysis involved five items or questions to determine a person’s TSB (the items are uploaded as supplementary materials). The first item assessed whether the participant had ever attempted to treat his illness. It was followed by items concerning when he started to seek treatment after the onset of the illness, the stage of the illness, the choice of treatment, and its course. Each item had options for answers ranging from 2 to 5, and they were scored accordingly. The total score for this domain was 15. Scores were summed up, and participants were given scores based on percentages. A score of 12 or more, which is equivalent to 80% or more, indicated appropriate TSB, and a score of less than 12, or equivalent to less than 80%, indicated inappropriate TSB. The scoring system was adapted from the original questionnaire [3].

For the qualitative portion, an interview guide was used. One of the research team members, who is a public health specialist, acted as a leader in organizing the data collection. He had been previously involved with qualitative research. Several training sessions were conducted with the research team to enhance his skills in qualitative methods. He had also attended some qualitative research workshops to improve his professional experience for this research. He conducted all the interview sessions and was assisted by a note-taker. The interview guide consisted of several open-ended questions that were directed towards the participants to initiate the discussion sessions. The contents of the interview guide included questions related to chronic illness and treatment choices, stages of seeking treatment, and barriers and experiences in seeking healthcare. Once the discussion flow got going, follow-up questions from the guide were asked concerning more specific aspects to gain more detail. All the interviews were audio-recorded.

## 4. Data Analysis

For the quantitative part, all data were entered and analyzed using IBM Statistics for Social Sciences (SPSS) version 24.0 software for Windows (IBM, Armonk, NY, USA). The data were checked and cleaned. The continuous variables were presented using mean and standard deviation (SD), whereas the categorical variables were described using frequency and percentage. The association between sociodemographic characteristics and inappropriate TSB were analyzed using simple and multiple logistic regression analysis. The findings were presented with crude and adjusted odds ratio (OR), a 95% confidence interval (CI), and *p*-value, and the level of significance was set at 0.05. For the qualitative portion, transcripts were analyzed using a thematic analysis approach [15]. Six steps were involved in the analysis, which included becoming familiar with the data, generating initial codes, searching for themes, reviewing the themes, defining the themes, and the write-up process. The primary researcher transcribed verbatim all the audio recordings of the FGD sessions. He and the other two research team members went through each transcript after every FGD to familiarize themselves with the data. Initial codes were generated independently by the three researchers. This was followed by the development of themes. The findings were compared and discussed, and a consensus was reached among the researchers before proceeding to the next transcript. The FGD session was continued until no new themes emerged from the analysis.

## 5. Results

### 5.1. Socio-Demographic Characteristics

All participants in the quantitative portion were males with a mean age of 41. The majority of the participants were Malays (89.2%), with secondary education being the majority form of education (53.8%). Most of the participants were married (85%) and had a monthly household income of less than RM 3100 (46.7%). The socio-demographic characteristics are presented in Table 1.

### 5.2. Treatment-Seeking Behavior 

Overall, 135 (35.4%) participants had appropriate TSB, and 246 (64.6%) participants had inappropriate TSB. The association between sociodemographic characteristics and inappropriate TSB using simple logistic regression analysis is presented in Table 2. Race, age, religion, education, marital status, and household income were significantly associated with inappropriate TSB at a univariable level. In multiple logistic regression analysis, three variables, including religion, household income, and marital status, were significantly associated with inappropriate TSB (Table 3). Non-Muslim participants had higher odds of engaging in inappropriate TSB compared to Muslim participants (OR 2.6; 95% CI: 1.1,6.7 *p* = 0.041). Those earning between RM 3100 and RM 6300 and those earning above RM 6300 had a higher probability of engaging in inappropriate TSB, compared to those earning <RM 3100 (OR 2.4; 95% CI: 1.5,3.8 *p* ≤ 0.001), (OR 4.5; 95% CI: 1.7, 11.6 *p* = 0.02). Lastly, those who are married had lower odds of engaging in inappropriate TSB compared to those who are single (OR 0.3; 95% CI: 0.1, 0.7 *p* = 0.035).

### 5.3. Reported Morbidities

The main morbidities reported by the participants were hypertension (26.5%) and diabetes mellitus (26.2%). Smoking seems to be a major concern, with more than half of the participants reporting smoking (57.7%). Based on TSB, reported morbidities are equally distributed among those with appropriate and inappropriate TSB (Table 4). There was no significant association between each reported morbidity and race, analyzed using chi-square test, with all the *p*-values > 0.05.

### 5.4. Qualitative Findings

A total of 42 male civil servants participated in the focus group discussions. All participants were Malay, except for one, who was Siamese. More than half of the participants (57.2%) were over 40 years old. The majority were married, with only five of them being single. As all of them are government servants, the minimum education status among the participants was secondary school, and the rest had completed tertiary education (61.9%). The thematic analysis identified one superordinate theme, which was treatment-seeking behavior, with six sub-ordinate themes. The sub-themes were: (1) health literacy; (2) stage of seeking treatment; (3) preference for alternative treatment; (4) perceived threat of illness; (5) self-treatment, and (6) the influence of family members and others. These themes were derived from the analysis of all six FGDs. Examples of their quotes are presented below.

### 5.5. Health Literacy 

Some of the participants developed a keen interest in health only after they were diagnosed with chronic diseases such as diabetes mellitus. They started reading about disease complications and other conditions in general. Several of the participants even possessed knowledge of how the medication works, which in turn helped them to seek proper treatment at the appropriate time. They also knew about illegal medications which were sold in Malaysia and in places such as Thailand. An example of the narration (in Malay, followed by an English translation) is provided below:

*“Kalau kat kerajaan, okey first step ambik hok mana dulu, pastu dia increase dose slow slow, ni kalau kat Thailand dia thump tinggi brapo gram selalu, jadi lepas tu memang la makan ubat kita tak jadi dah.” [“At Malaysian government facilities, medicines are gradually increased in dosage, where elsewhere in Thailand they prescribe a high dosage immediately so in the long run our body becomes resistant to it”]*

(HA, 57, FGD 01)

### 5.6. Stage When Seeking Treatment 

Stages when seeking treatment can be divided into early treatment-seeking or delayed treatment-seeking. Based on the findings from the FGD, the majority of the participants of all the FGD sessions belong to the delayed treatment-seeking category.

### 5.7. Delayed Treatment-Seeking

Many participants sought treatment only when they were severely sick, and they barely engaged in screening services and other forms of health services when they felt healthy. They also agreed that seeking treatment is disease-dependent. They also agreed that delayed TSB was due to attitude and behavior. This may refer to a lack of patience waiting at clinics, causing participants to opt to buy medicine at pharmacies. They claim that it is the natural attitude of men, asserting that men are impatient and would not spend time seeking treatment at clinics or hospitals. One of them stated the following:

*“Sebab sikap doctor, kade-kade rumoh pun kita ada first aid kan, ada ubat panadol ubat lain kade-kade..makan panadol dulu lah kalu sembuh okey kalu tok sembuh baru gi hospital, second chance plok”. [“It is due to attitude. Sometimes at home we have 1st aid kit and we have medication such as Panadol and others, so we take it initially and only if our condition is worsening then we go to hospital”].*

(HA, 57, FGD 01)

*“Dio sikap semulajadi lelaki, Sikap orang lelaki ini tak boleh tunggu lamo dia selalu cepat memberontak”. [“It is the natural attitude of men where men can’t wait for long and they easily retaliate.”]*

(HA, 57, FGD 01)

Another common trend among the participants was that they typically waited for more than 48 hours before seeking treatment. Apparently, doctors and friends told them that if the illness does not resolve after 48 hours, only then should a person visit a clinic or go to the hospital for treatment. One of the participants expressed the following:

*“biasanya makan Panadol dulu kat rumah, kalau berkesan ok la. Kalau tak ok baru gi klinik. Tapi jangan lebih 48 jam. Saya selalu tunggu 2 hari je, kalau dalam 2 hari tak ok, kena pergi klinik atau hospital la.” [“Usually, I take Panadol at home at the initial stages, if it’s effective then it’s ok. I usually wait for 2 days, if I’m still not ok within two days, then I go to the clinic or hospital.”]*

(RO, 56, FGD 04)

### 5.8. Early Treatment-Seeking

Only a few of the participants sought treatment immediately when they were not feeling well. One of the reasons was because they wanted to obtain a medical certificate if they went for treatment. In reality, this does not happen in all cases; the majority of them still go to work if they are capable, even if they have medical certificates with them. Some also firmly believe that prevention is better than a cure; thus, they would rather go early during the course of illness to seek treatment; one of them stated the following:

*“pencegahan awal lebih baik daripada rawatan bagi saya, biar sakit deme sikit pun kalu kita perlu kita dapat treatment, takut bende ni akan merebak kita tahu sekadar deme.” [“For me, early prevention is better than treatment, thus even if I’m having a mild fever, I immediately seek treatment, I am afraid it will spread and become worse.”] *

(HA, 57, FGD 01)

### 5.9. Preference for Alternative Treatment 

Traditional medication appears to play an important role in TSB among the participants. Most of them resort to traditional medications, such as the consumption of honey prior to visiting a healthcare professional. Most of them agreed that they only try simple traditional medications. However, when it comes to illegal medications that are sold in the market, they do not dare to take them, as they are afraid of their contents and side effects. One of the participants expressed the following:

*“Bagi saya bergantung kepada penyakit kita, kalu bo batuk gitu gak pade make madu.” [“For me it’s disease dependent, if it’s just mild cough then I will consume honey at first.”]*

(SA, 34, FGD 01)

According to one of them, this rationale in seeking treatment was based on the severity of the disease. If it is deemed severe, then he will seek treatment from a healthcare professional; otherwise, if it is simply a normal cough, he will merely take traditional medication such as honey. One participant said the following:

*“Majung, tonex daripada Indonesia gapo tok leh buat kiro..sebab kita tahu kesan dio tuh padahal steroid banyok gapo kito tok tahu buat mari Malaysia. Sebab tu ramai orang sempadan pergi dapat treatment alik siam, sebab apa..sebab bila dia pergi sekali sembuh, kalu ore klate panggil supo ambik tohok, ore sano dio pehe la..that why ramai ore sini gi ambik ubat alik siam, sebab ubat dio tak dok prekripsi dio bagi je hentam je sebab dio nok duit jah.” [“Medicines such as ‘Majung’ or ‘Tonex’ from Indonesia contains a lot of steroids. That is the reason many Malaysians go across the border to seek treatment. They get cured because of the high dosage of drugs used as they do not follow any specific guidelines.”] *

(HA, 57, FGD 01)

Some of the participants opted for traditional medication as the first line of treatment in combatting their illness. This method of treatment is adopted according to beliefs which are passed on for generations, and many of them do not know whether it is effective. In most scenarios, participants seek help from traditional healers first before seeking professional healthcare if their condition is still not improving. According to cultural beliefs, certain foods are considered to contain “wind,” which can cause pain and other illnesses. The examples of food which can cause ‘wind’ is when carbonated drinks are consumed together with nuts. One of the participants expressed the following:

*“Tahun 2005 tak silapnya saya sakit urat, sakit pinggan pergi berurut urat perut ni ore kampong ngurut kae dio tekan kuat la balik rumoh meme tok leh nok nafas, sakit sapa tak sedar diri..terus hantar ke hospital.” [“In the year 2005, I had some muscle and hip pain so I went for a traditional massage. They performed it wrongly and I couldn’t breath and I had to be rushed to the hospital after that.”]*

(AM, 44, FGD 02)

Even when it involves accidental cases, some of the participants sought help from traditional healers known as *“bomoh patah*” first for massages and treatment. This is common, as many fracture cases in northeastern Malaysia are diagnosed late, typically after about one week of injury. One of the participants stated the following:

*“Dulu saya pernah accident, mula-mula saya tidak pergi klinik dulu. Saya pergi mengurut dulu, tengok dalam tempoh 2 minggu masih sakit then baru pergi klinik buat xray tengok tulang dah patah actually.” [“I had an accident in the past, I didn’t go to the clinic initially. I tried traditional massage for about 2 weeks and only after that I went to clinic for X-ray as the pain was unbearable.”] *

(ZU, 34, FGD 05)

### 5.10. Perceived Threat of Illness 

Most of the participants had the urge to visit a healthcare professional only if a disease was considered severe; otherwise, they resorted to traditional medication as per cultural beliefs. One participant stated the following:

*“Biase kalu deme dengan batuk ni sebab antibody kita lah, kalu batuk hari ni esok hile tu biaso lah, deme tu meme deme bungkuh, deme ketar.’ [“Usually if it’s just cough and mild fever it’s just our antibodies trying to fight of the illness. If we get it will go off in a day or two unless its severe fever involving chills and rigors, then we have to go to hospital.”]*

(RO, 32, FGD 01)

To most participants, the deciding factor when selecting avenue for seeking treatment depended on their own rating of the severity of the disease. For illnesses which they consider “light,” such as hip pain, they would either self-treat or seek traditional medications first before visiting clinics or hospitals. Many of the participants also felt that being symptom-free can be equated with being healthy and having no illness at all. They waited for symptoms to appear to seek treatment or health advice. One participant stated the following:

*“ada pening-pening tu baru bulih pergi, buat maso loni keadaan tu terkawal lagi..tok payoh gi.” [“Only when having symptoms such as giddiness then I need to go and check. For now, everything is under control and I am free of symptoms so I don’t need to go.”]*

(GH, 48, FGD 02)

### 5.11. Self-treatment 

Many participants, based on cultural beliefs and understandings of disease severity, preferred to self-treat prior to engaging with proper healthcare services. This includes self-medication, such as paracetamol, etc. Participants also preferred to buy medications over the counter at pharmacies for illnesses which they regarded as mild and because over-the-counter medications are easy to obtain. One of the participants expressed the following:

*“Kalau sakit perut saya beli ubat dekat farmasi je.” [“If I have stomach pain then I just buy medication at the pharmacy.”]*

(HA, 57, FGD 01)

Participants also seemed to self-treat based on symptoms and myths. They employed methods such as drinking more water and getting more rest to recover from their illness. They believed that body signs and symptoms such as dark-colored urine are indicative of a lack of water in the body. One participant mentioned the following:

*“Kalau kurang air, minum air lebih sikit, kita tengok air kencing kita tu kalau pekat kita tau la, kurang air dalam tubuh, so kita minum air lebih sikit. Kalau rasa kurang tido, tidur lebih sikit.”[“I self-treat based on symptoms of the body. If the urine is dark-coloured, then I drink more water as I know there is insufficient water in the body.”]*

(AZ, 46, FGD 03)

Others resorted to buying medications over the counter at pharmacies. They used this as a first line of treatment, and only if symptoms were worsening, they went to a clinic or hospital. One participant indicated the following:

*“pergi farmasi beli ubat dulu la doc. Tak tahan jugak baru gi klinik.” [“I self-treat by buying medicine from the pharmacy initially, only then if it gets severe then I go to clinic.”]*

(KH, 45, FGD 04)

### 5.12. Influence of Family Members and Others

Although no persuasion was necessary for a small number of participants to seek professional healthcare services, the others required persuasion from their spouse or friends. It was also surprising to hear that several participants actually stated that they are earlier in seeking treatment compared to their wives. In Kelantan, the influence of village people, particularly the head of the village, plays an important role in TSB. Friends also influence TSB through experience-sharing, which influences decision-making related to treatment-seeking. Examples of their quotations are as follows:

*“Pengaruh orang kampong pun ada jugak, penghulu owh, pergi klinik tu la sembuh tengok cucuk apa, psikologi sungguh, psikologi belako.” [“There is influence from the village folks, such as the head of village. They recommend certain clinics and the persuasion and psychological factor really works.”]*

(HA, 57, FGD 01)

*“sini kita dah berkongsi, doctor nampok? Ini la yang berlaku sebenarnya, dekat sini bila kita tanyakan pendapat kita akan meluahkan pendapat masing-masing yang itu akan mempengaruhi keputusan kita.” [“In this group discussion, we are already sharing information. This is what is really happening when we ask for opinion then everybody will voice out their thoughts. This is what which will influence our decision making.”]*

(HA, 37, FGD 01)

Some of the participants also suggested that it is part of northeastern Malaysian culture to enjoy discussing and talking with friends and asking for opinions from one another. They are also more comfortable discussing health issues with friends rather than the doctor. This suggests that the participants believe and trust their friends more than the doctors and that friends are used as a platform to discuss health issues. One participant expressed the following:

*“kebanyakan la dia akan pergi ke satu tempat dia akan berbincang, macam saya sendiri tengok, dia akan tanya pendapat kawan dia..semalam saya sakit sini ghini, ada pendapat dok?(ceritakan situasi)..kawan-kawan dia yang akan bagi suggest, dia lebih selesa berkongsi dengan rakan-rakan terlebih dahulu daripada berjumpa dengan doctor..tanya pendapat rakan-rakan.” [“Most of us will discuss with friends regarding our health conditions. Based on the symptoms, friends will give suggestions on the proper treatment. Here you can see that men are more comfortable to discuss their problems with other friends 1st before seeking help from doctors.”]*

(HA, 37, FGD 01)

Some of the participants agreed that they need to be persuaded by family members to see a doctor. They felt too lazy to wait for long hours in a clinic when their perceived disease severity was low, so they preferred not to visit the doctor unless forced by their family members. Most of them agreed that the most significant persuasion typically comes from their spouse, who pesters them to go to clinics to seek treatment. Love and affection frequently provided motivation to seek treatment when not feeling well. One participant stated the following:

*“Memang isteri bising la. Bila ada orang worried pasal kita tu baru kita pergi.”([“I need to be persuaded by my wife to seek treatment. Only when someone is worried of us then we have the urge to go.”]*

(ZU, 34, FGD 05)

## 6. Discussion

Identifying the proportion of inappropriate TSB and exploring TSB experience among males is important to improve men’s health services, and subsequently improve their health status and reduce morbidity and mortality. In Malaysia, men’s health is gaining more attention. However, little is known about TSB among Malaysian men. As men’s inappropriate TSB leads to the under-utilization of available services and facilities in health care, this study was initiated to explore their experiences related to TSB. We found that TSB among male civil servants in northeastern Malaysia was poor and that factors contributing to it were multidimensional. TSB was influenced by health literacy, stage when seeking treatment, preference for alternative treatment, the perceived threat of the illness, preference to self-treat, and the influence of family members and others.

Several limitations should be considered before discussing our study findings. Firstly, a questionnaire was used, and most of the information obtained from the participants, particularly regarding the experience of disease and treatment duration, required them to recall prior information. Thus, this study was liable to being affected by recall bias. Recall bias was minimized as much as possible by establishing the requirement that one’s illness must have taken place within six months of the study for one to participate in the study. With this, the recall bias was reduced.

Furthermore, this study only included male civil servants. It prevents the generalization of findings to the general male population in northeastern Malaysia. Being in the civil services, they might be exposed to more information and access to health services, particularly government health services. However, findings from this study can be used as a reference to understand TSB among subgroups of the male population of Malaysia, particularly due to the limited information on this issue. Malaysia has a high number of civil servants; thus, this study would provide a better mean to understand their experiences and barriers in seeking healthcare to further improve the system in the future.

Consistent with the findings in other studies around the globe, most men had engaged in inappropriate TSB [3,4]. They did not treat their illness, delayed their treatment, did not utilize the proper channels to obtain treatment, or did not complete their treatment course. In a study performed in Uganda, men were also reluctant to seek treatment when struck by illness, and 63% of men had engaged in poor TSB [4]. In another study conducted in Iran, 68% of the general population had engaged in poor TSB [3]. These two findings are identical to our study, as most of the socio-demographic features were identical. These include socio-economic status and access to healthcare, since both countries had free government-funded healthcare systems and a private healthcare system; another common element was that Islam was the religion of most of the participants. 

In contrast, 22% of the men in Karachi had engaged in poor TSB and would never visit a healthcare facility [13]. In countries such as Pakistan, the main decision-makers in the households were the men. Men had more access to healthcare compared to women, and they did not need to consult anyone prior to seeking treatment. Despite this, the relatively low proportion of inappropriate TSB among the men in Karachi was partly because even self-treatment and visits to traditional healers were considered appropriate treatment-seeking in their study.

Our study found that if a disease was considered to be severe, only then did they seek proper medical care. Otherwise, they would resort to self-medication or see a traditional healer. The deciding factor regarding selecting the avenue for seeking treatment depended on their own rating of the severity of the illness. This was also demonstrated in another study, in which when a disease was perceived as being severe, only then would a person consult a healthcare professional. It also stated that participants of the study only considered the idea of consulting a doctor when encountering life-threatening situations. Illnesses such as body weakness and arthritis were considered to be mild illnesses, and people preferred to buy medications over the counter. The study also noted that certain illnesses such as diabetes and high blood pressure were perceived as non-treatable diseases [2,16].

In a qualitative study conducted in Malaysia, men typically equated masculinity with having a good body shape, being respected, having success with women, being a family man, and wielding economic power. In relation to health, they believed that men would not be respected if they were discovered to be ill, as illness would make them appear weak and vulnerable [17]. They thought that nothing could harm them and that they could withstand any disease or health-related issues; hence, men tended to seek treatment at later stages of illness when it was sometimes too late for treatment [18]. Similar findings were noted in this qualitative study, in which participants described themselves as strong and able to withstand any illness without needing to visit a doctor. They expressed they would bring their child or wife when they were ill, but they themselves would not seek treatment because they are strong adults and have experienced the illness before. 

In addition, the influence of family members and others played a vital role in TSB among men. Frequent persuasion from spouses and children made men seek treatment at an earlier stage. Those with family and job responsibilities engaged in better TSB; more specifically, participants revealed that they had to be responsible for the entire family. They could not afford to fall sick and miss work; therefore, they sought early treatment when they were ill. The same applies to those who held high positions in government jobs, wherein they could not transfer their work to someone else. They had to go to work even if they were ill; therefore, they preferred to seek treatment at early stages. A study in Malaysia demonstrated that men valued family values and advice from friends and family; hence, they adopt a healthy lifestyle and engage in positive TSB when they have a family [18,19].

Regarding self-treatment, most men preferred to self-treat prior to engaging with proper healthcare services. Self-treatment includes the purchase of medications over the counter without prescriptions and consuming spare medications available at home. According to a qualitative study, self-treatment was employed as a first line of treatment among men with acute illness, with about 55% of participants reporting having used self-medication as a first line of treatment when ill [20]. Another study in the UK uncovered that men did not want to endure the hassle of arranging an appointment with the doctor; thus, they preferred to self-treat first before seeking professional help [21].

According to a study conducted in Nigeria, the availability of medication over the counter influenced TSB, as medication was cheaper at pharmacies, and one would not need to pay consultation fees to a doctor, and this would save them a substantial amount of money [2]. Additionally, alternative medications which were available also encouraged men to engage in inappropriate TSB. Though men did not use alternative medicine as much as women, when they did use it, they used it more frequently than women [22].

A nationwide study conducted in Malaysia showed that it was easy to purchase over-the-counter medications without any prescriptions in the country. Medicines are cheaper at pharmacies relative to clinics and hospitals, and the ease of obtaining it would encourage people to purchase medications from the pharmacies rather than consulting a doctor. This factor would contribute towards poor TSB among the population. The study highlighted that men and elderly people bought more musculoskeletal, respiratory system, gastric, and anti-infective medication for systemic use, although these should be prescribed by a doctor. Without proper diagnosis, gastric pain may mask the symptoms of cardiovascular problems, and this poor TSB might lead to an increase in cardiovascular problems [23].

Our study also found that men engaged in delayed treatment-seeking due to several factors, such as the waiting times at government facilities being too long, poor appointment systems, having to take the day off to seek treatment, and the limited availability of doctors. Long waiting time was the main concern noted by everybody in the FGD sessions. Long waiting times at government clinics led to poor TSB among the participants. Firstly, they did not want to seek consultations due to the long waiting period, and, as a result, they preferred to withstand their illness for two to three days; only if it became worse would they seek treatment. Furthermore, participants said that even if some of them did not mind the long waiting period, it was not worth it, as the consultation with the doctor or the time spent with the doctor was less than five minutes. It is a “touch and go” system with the doctors, who are racing against time to see the enormous number of patients every day. 

The problem with the public primary healthcare in Malaysia is that it is overloaded with patients and understaffed. This results in long waiting hours at clinics and a lack of doctors to see patients. According to the National Audit Department in 2019 which was supported by the director-general of health, the public healthcare sector in Malaysia is underfunded, understaffed, underpaid, overworked, overstretched, and characterized by facilities overcrowded with patients. A review of the Malaysian healthcare system stated that the public healthcare system caters to about 65% of the population but is only served by 45% of all registered doctors [24]. Here, it is evident that despite almost 3000 health and community clinics throughout the country, public healthcare in Malaysia is still understaffed. Furthermore, many health clinics are still not computerized, and this causes massive delays in overall time spent at clinics. 

Other barriers which led to delayed treatment-seeking included attitudes of men, specifically that men were generally impatient and could not wait long at clinics, reluctance to engage in screening services, and inadequate knowledge or health illiteracy. A study conducted in Africa yielded a similar finding, wherein men were reluctant to undergo health screening, as they were afraid to discover what they do not know about their health. Once they learned about a disease which they had, they feared being stigmatized or isolated by other people, as men are supposed to appear strong and free of illness [20]. Another study conducted in the UK stated that the reason for delayed treatment-seeking among men was that men ignored signs of ill health, assuming their condition will improve without the need of medical assistance [21]. This again demonstrates the attitude of men who perceive themselves as strong and having a high ego which prevents them from seeking treatment. 

Another important finding from our study was the preference for alternative treatment. Traditional medicine plays a pivotal role in Malaysian culture. Usually, traditional medication was selected as the first line of treatment among most of the participants. This method of treatment is pursued according to beliefs which are passed on for generations. Traditional medications include taking herbs and herbal products, seeking treatment from a traditional healer, and seeking treatment from religious sources such as the recital of Al-Quran or consuming holy water. 

According to a study performed in 2009 in Malaysia, the use of traditional and complementary medicine in the country was high, with a reported prevalence of almost 62%, with the highest being among Malays. It was reported that biologically based therapies, which include herbal therapy, were most commonly used for health problems (89%) and for health maintenance (88%) [25]. The Ministry of Health in Malaysia has its own division for overseeing traditional and complimentary medicine (TCM) practice in the country. 

A more gender-sensitive approach should be used to tackle men’s health issues in Malaysia. The need has come for a specialized men’s clinic. Due to various dilemmas faced by men in utilizing primary healthcare services in Malaysia, a dedicated clinic should be implemented in all districts in Malaysia. A men’s-only clinic run by an all-male healthcare team, as suggested by the participants in this study, might not be feasible. However, providing well-trained and gender-sensitive staff in aspects related to men’s health are important, as well as being able to provide medications and specialist care treatment for men. Another recommendation would be to cater more towards men’s needs rather than asking men to adapt to the present healthcare system. With the current advancement in information technology, men prefer to resort to the internet to seek information regarding health. An online health service portal should be made available for Malaysian men, to allow them to seek consultation and arrange an appointment to see a doctor. Men generally prefer this approach, as it is seen as being more confidential, and it is a hassle-free process. 

In general, men do admit their susceptibility to ill health, but they often feel restricted in their ability to admit illness due to the importance of masculinity among men. Talking about health problems is more acceptable among women and is an unwelcome topic for men. This may be the main reason that men tend to shy away from health services. On the other hand, the traits of masculinity demand that men take charge and be personally active in their response to ill health, without the interference of a healthcare professional. Therefore, they prefer methods such as self-monitoring, and the internet seems to be their best choice. Men were able to understand their illness better, and it is a good means to deal with their illness without the physical presence of a healthcare provider. It maintained their anonymity, and men were more readily willing to accept this form of help-seeking, as it was still considered self-controlled [26]. Based on this evidence, the need to provide a specialized online health service is essential so that men can find accurate, precise and, most importantly, credible information on health matters. The current information available on the internet are from varied sources and are often misleading. 

Addressing men’s health issues in Malaysia is long overdue. Problems faced by men in seeking healthcare are not new, but only recently did this begin to gain attention in Malaysia and around the globe. With the recent launch of the National Men’s Health Plan of Action 2018–2023, MOH should use this as an opportunity to improve the status of men’s health in Malaysia. 

## 7. Conclusions

Majority of male civil servants in North-eastern Malaysia engaged in inappropriate TSB. There were multiple contributing factors, which included health literacy, stage of seeking treatment, preference for alternative treatment, the perceived threat of illness, preference to self-treat and influence from family members and others. This study provided new valuable evidence on men’s TSB in North-eastern Malaysia. The findings can be used to facilitate and improve current policies and implementation of men’s health services throughout the country.

## Figures and Tables

**Table 1:** Sociodemographic characteristics of participants (*n* = 381).

Variable	Frequency (%) ^a^,
Age (year)	41 (10.1) ^a^
21–40 (youth)	193 (50.8)
> 40	187 (49.2)
Race	
Malay	340 (89.2)
Indian	19 (5.1)
Chinese	12 (2.1)
Siamese	8 (3.1)
Others	2 (0.5)
Religion	
Islam	341 (89.5)
Buddha	18 (4.7)
Hindu	19 (5.0)
Christian	3 (0.8)
Education	
Primary	11 (2.9)
Secondary	205 (53.8)
Tertiary	165 (43.3)
Marital Status	
Single	47 (12.3)
Married	324 (85.0)
Divorcee//Widower	10 (2.7)
Household Income (RM)	3621 (2016) ^a^
<3100 (B40)	178 (46.7)
3100–6300(M40)	170 (44.6)
> 6300	33 (8.7)

^a^ mean(SD).

**Table 2:** Association between sociodemographic characteristics with inappropriate TSB, using simple logistic regression analysis (*n* = 381).

Variable	B ^a^	S.E.^b^	Crude OR ^c^ (95%CI ^d^)	*p*-Value
Race				
Malay	0	-	1	
Indian	1.6	0.8	5.2 (1.2,22.8)	0.003
Siamese	1.4	1	4.2 (0.5,35.1)	0.128
Chinese	0.6	0.7	1.8 (0.5,6.8)	0.307
Age (year)				
21–40	0	-	1	
> 40	0.3	0.2	0.7 (0.4,0.9)	0.028
Religion				
Muslim	0	-	1	
Non-Muslim	0.5	0.1	3.4 (1.4,8.4)	0.011
Education				
Primary	0	-	1	
Secondary	1.4	0.7	4.1 (1.0,16.2)	0.004
Tertiary	1.9	0.7	6.7 (1.7,26.3)	0.021
Marital Status				
Single	0	-	1	
Married	0.7	0.4	0.4 (0.2,0.8)	0.013
Divorced/widower	0.9	0.8	0.6 (0.1,2.5)	0.454
Household Income (RM)				
<3100	0	-	1	
3100–6300	0.7	0.2	2.1 (1.4,3.3)	<0.001
>6300	1.3	0.5	3.7 (1.4,9.5)	0.015

^a^ B = unstandardized regression; ^b^ S.E. = standard error; ^c^ OR = odds ratio; ^d^ CI = confidence interval.

**Table 3:** Multiple logistic regression analysis for the association between sociodemographic characteristics and inappropriate TSB (*n* = 381).

Variable	B ^a^	S.E.^b^	Adj. OR ^c^ (95%CI ^d^)	*p*-Value
Religion				
Muslim	0	-	1	
Non-Muslim	0.9	0.5	2.6 (1.1,6.7)	0.041
Household Income (RM)				
<3100	0	-	1	
3100–6300	0.8	0.2	2.4 (1.5,3.8)	<0.001
>6300	1.5	0.5	4.5 (1.7,11.6)	0.022
Marital status				
Single	0	-	1	
Married	−1.1	0.4	0.3 (0.1,0.7)	0.035
Divorcee//Widower	−0.7	0.8	0.4 (0.1,2.1)	0.373

^a^ B = unstandardized regression; ^b^ S.E. = Standard error; ^c^ Adj.OR = Adjusted Odds Ratio; ^d^ CI = Confidence interval; Constant −4.528; Forward Likelihood Ratio applied; No multicollinearity and no interaction detected; Hosmer-Lemeshow test, *p*-value = 0.202; Classficiation table 80% correctly classified; Area under the receiver operating characteristic was 85.5%.

**Table 4:** Reported morbidities among participants based on TSB (*n* = 381).

Variable	Total	Appropriate TSB	Inappropriate TSB
	Frequency (%)	Frequency (%)	Frequency (%)
Hypertension			
Yes	101 (26.5)	44 (32.6)	57 (23.2)
No	260 (68.2)	87 (64.4)	173 (70.3)
Don’t know	20 (5.3)	4 (3.0)	16 (6.5)
Diabetes Mellitus			
Yes	100 (26.2)	36 (26.7)	64 (26.0)
No	261 (68.5)	95 (70.3)	166 (67.5)
Don’t know	20 (5.3)	4 (3.0)	16 (6.5)
Smoking			
Yes	220 (57.7)	79 (58.5)	141 (57.3)
No	161 (42.3)	56 (41.5)	105 (42.7)
Sexual Problems			
Yes	54 (14.2)	15 (11.1)	39 (15.9)
No	319 (83.7)	116 (85.9)	203 (82.5)
Don’t know	8 (2.1)	4 (3.0)	4 (1.6)
Mental Health Problems			
Yes	26 (6.8)	5 (3.7)	21 (8.5)
No	353 (92.7)	129 (95.6)	224 (91.0)
Don’t know	2 (0.5)	1 (0.7)	1 (0.5)
Obesity			
Yes	106 (27.8)	31 (23.0)	75 (30.5)
No	248 (65.1)	93 (68.9)	155 (63.0)
Don’t know	27 (7.1)	11 (8.1)	16 (6.5)

## Supplementary Materials

The following are available online at https://www.mdpi.com/1660-4601/17/8/2713/s1, Questionnaire S1: Treatment seeking Behaviour.
