# Supplementary material for: Treatment-Seeking Behavior Among Male Civil Servants in Northeastern Malaysia: A Mixed-Methods Study
Source: Int J Environ Res Public Health. 2020 Apr 15;17(8):2713. doi: 10.3390/ijerph17082713 (PMC7216173; doi:10.3390/ijerph17082713)
Supplement: Supplementary file 1 [file ijerph-17-02713-s001.pdf]

MALAY VERSION

BORANG SOAL-SELIDIK PERILAKU MENDAPATKAN RAWATAN

PERILAKU MENDAPATKAN RAWATAN

Soalan ini merujuk kepada **penyakit akut** yang telah anda alami dalam tempoh 6 bulan yang lepas yang telah menjejaskan prestasi anda di tempat kerja.

1) Adakah anda pernah cuba merawat penyakit anda?
   A) Ya
   B) Tidak – jika tidak, anda tidak pelu menjawab soalan 2 hingga 5

2) Berapa hari selepas mendapat tanda-tanda penyakit, anda mendapatkan rawatan daripada pengamal perubatan?
   A) Pada hari yang sama
   B) Keesokan hari
   C) 2 hari kemudian
   D) 3 – 7 hari
   E) > 7 hari

3) Pada peringkat penyakit yang manakah anda pergi berjumpa dengan pengamal perubatan?

   A) Pada peringkat awal dan tanda-tanda penyakit berada ditahap ringan
   B) Pada peringkat serius penyakit

4) Manakah tempat yang pertama anda pergi untuk mendapatkan rawatan?

   A) Klinik kerajaan / klinik swasta/ hospital kerajaan/ hospital swasta
   B) Farmasi
   C) Pengamal perubatan tradisional
   D) Saya tidak menggunakan perkhidmatan yang dinyatakan di atas, saya merawat diri sendiri di rumah

5) Sehingga peringkat manakah anda sempurnakan rawatan anda?

   A) Lengkap rawatan sepenuhnya
   B) Tanda penyakit telah tiada tetapi tempoh rawatan masih belum lengkap

ENGLISH VERSION

Treatment seeking Behaviour

1) Do you have attempted to treat your illness?
   C) Yes
   D) No – If no, you do not need to answer questions number 2 to 5

2) How many days after the onset of disease symptoms did you seek treatment from a healthcare provider?
   A) The same day
   B) The next day
   C) 2 days later
   D) 3-7 days
   E) > 7 days

3) At what stage of your disease did you go to a healthcare provider?
   A) In early stage and mild onset of symptoms
   B) In serious stage of disease

4) Where did you go for treatment in the first step?
   A) Government clinic/ Private clinic/ hospital
   B) Pharmacy
   C) Traditional healers
   D) I have not referred to any of the above, I usually just self-treat at home

5) Until which step did you complete your course of treatment?
   A) Complete full course of treatment
   B) Symptoms resolved but do not complete course of treatment
